# Notices

**Published:** 1869-10

**Authors:** 


### NOTICES.

London Lancet is published monthly, simultaneously in London and by Wm. C. Herald, No. 32 Beekman St., New York, for $5 a year, postage 24 cents per annum; it is considered as "authority" in the medical world, and is commended to the profession throughout the country as a reliable medium of authentic medical news and progress.

Mental Cure, illustrating the influence of the mind on the body, both in health and disease, and the method of treatment, by W. F. Evans, published by the Messrs. Carter 25, Broomfield St., Boston, 64 pp. sent for $2 post-paid.

The Trance or Pathetism and correlative phenomena of Laroy Sunderland, published by James Meeker, Chicago, Ill. and sent post-paid for $1 50. 407 pp. 12mo.

The Rev. Dr. Willoughby and His Wine.—This is the title of a new book, just published by the National Temperance Society, of 458 pages, written by Mrs. Mary Spring Walker. Illustrated with four choice engravings from original designs, and will be sent to any address on receipt of price, $1 50. Address J. N. Stearns, Publishing Agent, 172 William St., N. Y.

The Dynamic Cure of Laroy Sunderland, third edition. 216 pp. 12mo, published by James Walker, Chicago, Ill. 1869. Treating of the Origin of Disease, Natures, Methods, Common Fallacies, Nutrition, etc., etc.

Patty Gray's journey to the Cotton Islands is a beautiful little book by Caroline H. Dall, designed for children, is published by Lee & Shepard, Boston, Mass. 201 pp., sent post-paid for sixty cents. It is instructive and entertaining to the old as well as the young. The same enterprising House has published "An American Woman in Europe," being the journal of two and a half year's sojourn in Germany, Switzerland, France and Italy. 338 pp., 12mo. This entertaining book may be profitably read by all who have travelled through those countries and will revive many pleasant memories of things seen and done; it is truthful, practical and just.

"Aunt Dinah's Pledge," by Miss Mary Dwinell Chellis, author of the Temperance Doctor, is one of the very useful and entertaining issues of "The National Temperance Society and Publication House," 172 William St., New York.

Man in Genesis and Geology, by Joseph P. Thompson,

D.D., LL. D. Published by S. R. Wells, 389 Broadway, New York. This book is valuable in that it treats of actual facts, fixed and ascertained. It does not deal in conjecture and mere supposition, hence may be profitably read, especially by every Bible student and by every geologist; it evinces great research, embraces a wide range of subjects and is reliable and definite in its statements, and merits a wide circulation. Price $1 by mail, free of postage.

WM. WOOD & CO. have published "ANAL FISSURE, its Aetiology, Pathology, Diagnosis and Treatment," by William Bodenhamer, A.M. M.D., author of Congenital Diseases of the Rectum, and Professor of Diseases of the Rectum and Contiguous Textures, 199 pp. 8vo., illustrated by numerous cases and drawings. The editor, after a personal acquaintance with the Author of more than thirty years, and a familiarity with his mode of practice, does not hesitate to pronounce Dr. Bodenhamer as the most successful living practitioner at home or abroad in ANAL FISSURE, FISTULA, STRICTURE of the bowels, PILES.

His manipulations performed in a manner peculiar to himself, are shown and communicated to the Profession with great courtesy and cheerfulness when called upon at his residence, 237 5th Ave., New York; he has not only obtained its confidence and respect here, but has drawn to himself an amount of practice from a list of patients of which he may well be proud, members of various state legislatures, governors, senators, officers in the Army and Navy, and from all the professions, and we feel that we are doing our readers a very great service in making these statements, (without the author's knowledge) in case any of them should need his skill in the direction referred to.

As the cases given are very instructive to the general reader, and the causes of these most distressing diseases are plainly stated, and by knowing them the maladies may be avoided, we will keep the book for sale in our office, or will send it post-paid on having $2.25 remitted in a post office order, for safety, to address of HALL'S JOURNAL OF HEALTH, 176 Broadway, New York.

